# The role of ATP-binding cassette subfamily A in the etiology of Alzheimer’s disease

**DOI:** 10.1186/s13024-022-00536-w

**Published:** 2022-04-27

**Authors:** Liene Bossaerts, Rita Cacace, Christine Van Broeckhoven

**Affiliations:** 1 Neurodegenerative Brain Diseases Group, VIB Center for Molecular Neurology, Antwerp, Belgium; 2 Department of Biomedical Sciences, University of Antwerp – CDE, Universiteitsplein 1, B-2610 Antwerp, Belgium

**Keywords:** ATP-binding cassette transporter, ABCA1, ABCA2, ABCA5, ABCA7, Alzheimer’s disease, Amyloid β, Cholesterol homeostasis

## Abstract

**Background:**

Alzheimer’s disease (AD) is the leading cause of dementia, clinically characterized by memory deficits and progressive cognitive decline. Despite decades of research effective therapies are lacking, and a large part of the genetic heritability remains unidentified. *ABCA7* and *ABCA1*, members of the ATP-binding cassette subfamily A (ABCA), were identified as AD risk genes in genome-wide association studies. Nevertheless, genetic and/or functional studies propose a link between AD and two other members of the ABCA subclass, i.e., ABCA2 and ABCA5.

**Main body:**

Changes in expression or dysfunction of these transporters were found to increase amyloid β levels. This might be related to the common role of ABCA transporters in cellular cholesterol homeostasis, for which a prominent role in AD development has been suggested. In this review, we provide a comprehensive overview and discussion on the contribution of the ABCA subfamily to the etiopathogenesis of AD.

**Conclusions:**

A better understanding of the function and identification of disease-associated genetic variants in ABCA transporters can contribute to the development of novel therapeutic strategies for AD.

## Background

Alzheimer’s disease (AD) is the most common cause of dementia and represents the sixth-leading cause of death in the United States [1]. Age is the most important known risk factor for AD, with the risk approximately doubling every five years after the age of 65 [2]. While most patients have an onset age above 65 years (late-onset AD or LOAD), in 10% of the patients the disease manifests before the age of 65 years, known as early-onset AD (EOAD) [3]. EOAD is almost entirely genetically determined, with a heritability between 92 and 100% [4]. In the early 1990’s, genetic studies in large pedigrees with autosomal dominant inheritance patterns of AD, led to the discovery of pathogenic mutations in three genes: amyloid precursor protein (*APP*), presenilin-1 (*PSEN1*) and presenilin-2 (*PSEN2*) [5–7]. The discovery of these genes contributed significantly to the understanding of the disease and shaped the amyloid β (Aβ) cascade hypothesis. Neurotoxic Aβ peptides are the major component of senile plaques, an important pathological hallmark of AD, and are generated through the successive proteolytic cleavage of APP by β- and γ-secretases [8]. PSEN1 and PSEN2 represent catalytic subunits of γ-secretase [9]. Mutations in *APP* and the presenilins explain around 10% of the EOAD cases, leaving the majority of the patients genetically unexplained [4]. LOAD is a more complex disorder, with an estimated heritability of 58% to 79% [10]. The ε4 allele of the apolipoprotein E (*APOE)* gene is recognized as a strong risk factor for LOAD [11, 12]. In heterozygous carriers, the risk for developing AD is 3 to 4 times higher compared to *APOE* ε4 noncarriers and increases 9 to 15 times in homozygous *APOE* ε4 carriers [13]. Genome-wide association studies (GWAS) in large LOAD patient and control cohorts led to the identification of common variants associated with AD in numerous genomic risk loci, including two members of the ATP-binding cassette subfamily A (ABCA), *ABCA7* and *ABCA1* [14, 15].

## Main text

### ABC transporters and the A-subfamily

**Fig. 1:**
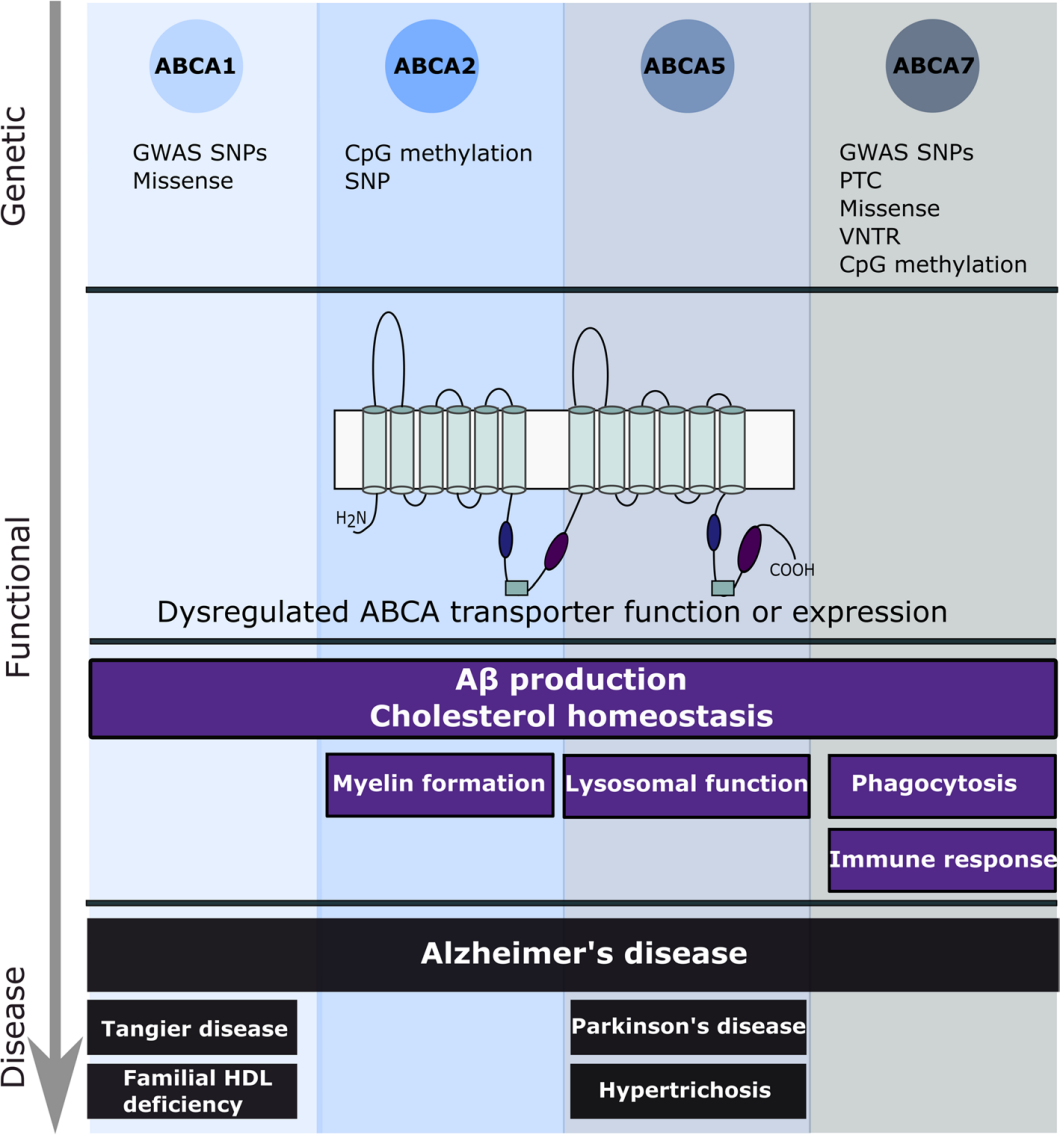
Overview of genetic variants and functional mechanisms involved in the etiopathology of ABCA-transporters ABCA1, 2, 5 and 7 to Alzheimer’s disease. Other diseases in which the described ABCA transporters are implicated are also shown

The ATP-binding cassette (ABC) transporter family is a superfamily of highly conserved integral membrane proteins responsible for the transport of various substrates across cellular membranes. Based on amino acid sequence similarity and phylogeny, seven subfamilies from ABCA to ABCG are defined, which classify all 48 functional human ABC transporters [16]. ABC transporters share a characteristic architecture, consisting of four core domains: two nucleotide binding domains (NBD) and two transmembrane domains (TMD) (Fig. 1). The NBDs provide the energy for substrate transport by ATP-binding and ATP-hydrolysis and contain three highly conserved motifs: Walker A and B motifs and a signature (C) motif. The TMDs typically contain six membrane-spanning α-helices and provide a pathway across the membrane for substrate transport [17]. These domains also harbor ligand-binding sites that determine the substrate specificity [18].

The ABCA subfamily comprises 12 functional transporters, ABCA1 to ABCA13, with ABCA11 representing a transcribed pseudogene. The A-subfamily is characterized by two large extracellular loops between the first and second helix of each transmembrane domain, which can function as ligand binding sites (Fig. 1) [19]. Several members have been identified as lipid transporters in different body locations [20]. The subfamily can be divided in two subgroups, based on phylogenetic analysis and chromosomal location [17]. The first subgroup of five genes (*ABCA5*-*6* and *ABCA8*-*10*) is organized in a head-to-tail cluster on chromosome 17q24, while the second group of seven genes (*ABCA1*-*4*, *ABCA7* and *ABCA12*-*13*) is dispersed on six chromosomes [17].

Over the past years, A-subclass ABC proteins have gained a lot of attention due to their implication in human diseases. To date, mutations in five *ABCA* genes are causatively linked to monogenic recessive disorders: *ABCA1* (Tangier disease), *ABCA3* (neonatal surfactant deficiency), *ABCA4* (Stargardt disease), *ABCA12* (harlequin ichthyosis) and most recently *ABCA5* was linked to congenital generalized hypertrichosis terminalis [21–25]. Additionally, rare coding variants in *ABCA13*, increase the susceptibility to schizophrenia and bipolar disorder [26, 27]. Moreover, GWAS recognized *ABCA7* and more recently *ABCA1* as risk genes for LOAD [15, 28]. Post-GWAS genetic studies identified common and rare *ABCA7* variants that influence AD risk, establishing *ABCA7* as an important AD risk gene. Although the underlying mechanism linking *ABCA7* risk variants to AD pathogenesis is poorly understood, ABCA7 is functionally involved in several molecular processes linked to AD etiology.

Besides ABCA1 and ABCA7, two additional ABCA members, ABCA2 and ABCA5, have been genetically and/or functionally linked to AD, supporting a broader function of this protein subfamily to the etiopathogenesis of AD (Fig. 1). Interestingly, these ABCA transporters are all implicated in cholesterol homeostasis, a pathway for which an important role in AD has been suggested, as highlighted below. In this review, we will first discuss the link between cholesterol metabolism and AD, before reviewing the genetic and functional evidence linking ABCA1, ABCA2, ABCA5 and ABCA7 to AD.

### The link between cholesterol homeostasis and AD

Cholesterol is a key component of mammalian cell membranes and it is involved in a large number of cellular processes [29]. Membrane cholesterol regulates membrane fluidity, rigidity and permeability by interacting with surrounding bilayer lipids and regulates signal transduction by interacting with transmembrane proteins [29]. The brain contains the highest cholesterol levels in the body, and tight regulation of its synthesis, storage, transport and removal is essential for neuronal functioning [30]. Brain cholesterol mainly originates from de novo synthesis, since systemic lipoprotein uptake is prevented by the blood–brain barrier (BBB) [31]. In the adult brain, cholesterol synthesis is mostly dedicated to astrocytes, which then redistribute cholesterol to neurons, a process mediated by ABCA1 [32]. ABCA1 exports excess cellular cholesterol and phospholipids to apolipoproteins [33]. While apolipoprotein A1 (ApoA1) is the major component of high-density lipoprotein (HDL) particles in the plasma, shuttling cholesterol to the liver for excretion, ApoE is the main cholesterol transporter in the central nervous system and is predominantly produced by astrocytes [33]. In the brain, the HDL-like ApoE-cholesterol-phospholipid complexes can be internalized by neurons, by binding to cell surface receptors, such as the low-density lipoprotein (LDL) receptor [34]. Excess cholesterol can be excreted by conversion to 24-S-hydroxycholesterol, which can readily pass the BBB to be further metabolized by the liver or can be esterified and stored intracellularly as lipid droplets [35, 36]. In addition, it is hypothesized that brain cholesterol is eliminated through the BBB by efflux transporters, such as ABC transporters [37].

β- and γ-secretases mainly operate in cholesterol-enriched membrane microdomains termed lipid rafts, while α-secretase mainly localizes to non-raft regions. High plasma membrane cholesterol levels facilitate the colocalization of APP with β- and γ-secretases, promoting amyloidogenic APP processing and therefore Aβ production [38]. In line, cholesterol depletion promotes the nonamyloidogenic α-secretase cleavage of APP, leading to a reduced Aβ production [39]. Despite the separation of brain and peripheral cholesterol pools, epidemiological studies identified a link between high serum cholesterol levels and AD risk [40]. In parallel, the use of cholesterol-lowering agents, i.e. statins, is associated with lower AD risk [41]. The flux of plasma oxysterols towards the central nervous system following hypercholesterolemia, together with disruption of the BBB might explain the link between serum cholesterol and AD [42].

A first genetic link between AD and lipid metabolism was established when the ε4 allele of *APOE* was identified as a major genetic risk factor for AD and cerebral amyloid angiopathy (CAA) [43, 44]. ApoE ε4 is suggested to increase Aβ aggregation and decrease Aβ clearance [45, 46]. Indeed, ApoE colocalizes with senile plaques, neurofibrillary tangles, and vascular amyloid [12], and was found to bind Aβ, although the ApoE ε4 isoform shows a decreased Aβ binding affinity [47, 48]. In addition, an isoform-dependent difference in cellular cholesterol efflux is observed, with ApoE ε4 showing the least efflux capacity [49]. Decades after the identification of *APOE* ε4 as a strong AD risk factor, GWAS in LOAD cohorts identified a high number of risk genes that are implicated in lipid metabolism, including two genes of the ABCA subfamily: *ABCA1* and *ABCA7* (Fig. 1) [14, 50, 51].

### ABCA1

In the periphery, ABCA1 promotes the release of cellular cholesterol and phospholipids to lipid-poor apolipoproteins, mainly ApoA1, to generate HDL [52]. Since cholesterol is mainly catabolized in the liver, efflux of excessive cellular cholesterol by ABCA1 to ApoA1 plays a key role in the reverse cholesterol transport pathway in order to deliver HDL to the liver for excretion [53]. The identification of *ABCA1* loss of function mutations in patients with HDL-deficiency syndromes, including Tangier disease, confirmed the role of ABCA1 in cellular cholesterol homeostasis [54]. Tangier disease is a recessive disorder characterized by extremely low plasma HDL and ApoA1 levels, intracellular cholesterol depositions, premature atherosclerosis and peripheral neuropathy [54]. The role of ABCA1 in the periphery has been extensively studied. Nevertheless, *ABCA1* is highly expressed in the human brain, with the highest expression in neurons and microglia [55]. Studies in mice showed that in the central nervous system, ABCA1 is directly involved in brain cholesterol homeostasis by exporting cholesterol through the BBB [56]. In addition, loss of *Abca1* results in a major decrease in ApoE protein levels and ApoE lipidation, as well as an impaired hippocampal neurite morphology in mice, suggesting a role for ABCA1 in AD [33, 57]. Lipidation of ApoE is required for its functioning, including the ability to bind Aβ [48], and a lower lipidation status has been observed in ApoE ε4 compared to ApoE ε3 produced by human iPSC-derived astrocytes [58]. Furthermore, *Abca1* deficiency increases Aβ deposition as well as CAA in two AD mouse models [59, 60], and is linked to cognitive deficits in mice [57, 61]. Fitz et al. demonstrate that *Abca1* deficiency in an AD mouse model negatively impacts amyloid deposition, Aβ clearance and memory in mice expressing human APOE ε4 but not APOE ε3, suggesting an interaction between ABCA1 and other genetic risk factors [62]. In parallel, overexpression of *Abca1* in an AD mouse model reduced fibrillogenesis and deposition of Aβ in the brain, possibly related to the increased lipidation of ApoE [63]. Selective stimulation of *Abca1* with an ABCA1 agonist in mice expressing human APOE *ε*4, increased lipidation of ApoE ε4 and ameliorated ApoE ε4-driven cognitive impairments and brain pathology, rendering it to a similar level as the mice expressing ApoE ε3 [64]. In addition, ABCA1 membrane expression in mice primary astrocytes is diminished in cells expressing human ApoE ε4 compared to ApoE ε3 expressing cells due to a reduced ABCA1 recycling [65]. In parallel, a reduction in ABCA1 protein levels is observed in human astrocytes expressing ApoE ε4, possibly contributing to the ineffective cholesterol efflux in ApoE ε4 cells [66]. Upregulation of ABCA1 and the subsequent increase in APOE lipidation might present a potential therapeutic strategy to ameliorate AD-pathology driven by APOE ε4.

*ABCA1* is transcriptionally regulated by oxysterol-activated liver X receptors (LXRs), nuclear receptors which bind to DNA sequences of their target genes as heterodimers with retinoid X receptors (RXRs) to activate transcription [67]. Numerous studies have pursued the use of LXR or RXR agonists to reduce AD-related brain pathology and cognitive impairment, as recently reviewed by Fitz and colleagues [68]. Following LXR activation, a decrease in amyloidogenic APP processing and Aβ secretion has been demonstrated in vitro and in AD mouse models, and improvement of cognitive deficits has been observed in AD mice [69, 70]. These changes were associated with an increased ABCA1 expression and propose the induction of functional ABCA1 as a promising therapeutic option for AD [69, 70].

In vitro experiments with skin fibroblasts derived from two Tangier disease patients carrying homozygous *ABCA1* premature termination codon (PTC) or missense mutations leading to a loss of functional protein show an increased production of Aβ compared to control cells [71]. Interestingly, upregulation of *ABCA1* expression via a synthetic LXR ligand led to a further Aβ increase in cells carrying a missense mutation (N935S) and stayed the same in cells carrying a nonsense mutation, signaling that functional and full-length ABCA1 is required to benefit from the effect of LXR/RXR agonists on Aβ secretion [71]. This is in line with the clinical phenotype of the N935S patient, who had extremely low HDL levels and developed severe dementia and amyloid depositions by the age of 60 [71]. Another case with a relevant link with AD is a patient carrying a compound heterozygous mutation (D1099Y and F2009S) in *ABCA1*, who presented with low HDL but no cardiovascular disease, and later developed and died of CAA [72].

**Fig. 2:**
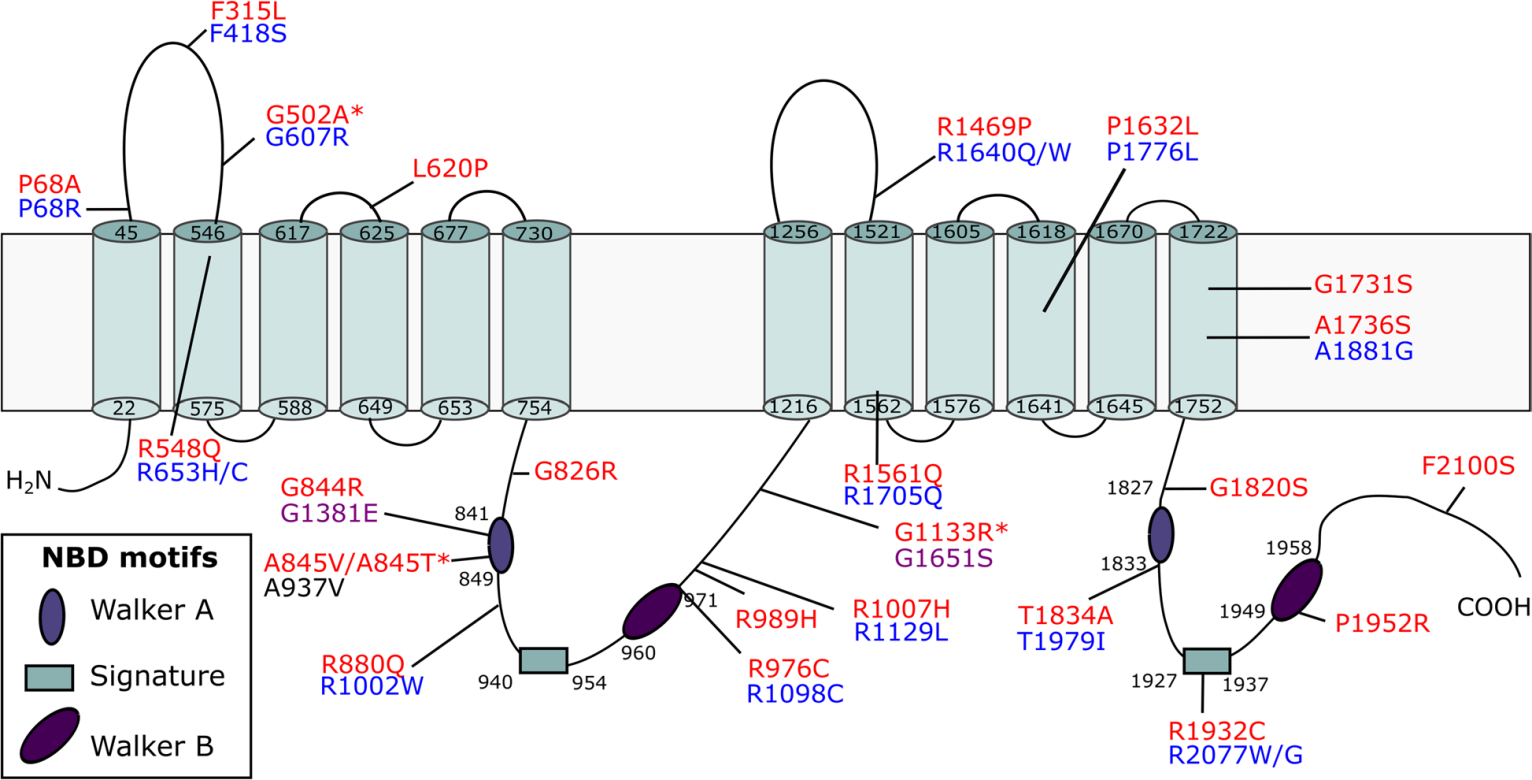
Topological model of ABCA7. Pathogenic *ABCA7* missense mutations leading to mislocalization and subsequent loss of functional protein as well as *ABCA7* missense mutations corresponding to pathogenic mutations in ABCA transporters implicated in human disease are shown. The ABCA7 sequence was aligned with sequences of ABCA1, 3, 4, 5 and 12. Pathogenic missense mutations in these five genes were downloaded from the ClinVar database (https://www.ncbi.nlm.nih.gov/clinvar/). ABCA7 missense mutations, previously reported by Le Guennec et al., Sassi et al., Bellenguez et al., De Roeck et al., and Bossaerts et al., that correspond to pathogenic missense mutations in ABCA1, 3, 4, 5 and 12 are shown in the figure [78–82]. Variants marked with a ‘*’ were identified in control individual(s) only. Protein domain and motif information was based on alignment with ABCA1 [83, 84]. *ABCA7* missense variants are shown in red. Corresponding pathogenic *ABCA1*, *ABCA4* and *ABCA12* mutations are shown in black, blue and purple respectively

The *ABCA1* gene is located near a linkage peak on chromosome 9, previously identified through genome-wide AD linkage studies, and is a good candidate gene given its function in cholesterol homeostasis [73, 74]. Since the early 2000’s, 20 studies exploring the association of *ABCA1* common single nucleotide polymorphisms (SNPs) with AD have been published, reporting conflicting results (PubMed, accessed 20 September 2021). An established ABCA1 loss-of-function mutation involved in familial HDL-deficiency, N1800H, is associated with low ApoE plasma levels and a higher risk for AD and cerebrovascular disease [75]. In a family with 4 AD patients, co-segregation of a missense variant (rs137854495; p.A937V) with AD was reported [76]. This variant was previously identified in Tangier disease patients as part of a compound heterozygous mutation [25]. The mutation resides in the Walker A motif of the first NBD and abolishes cholesterol efflux [77]. Interestingly, the same conserved Alanine to Valine substitution in ABCA7 (p.A845V) was identified in a patient with AD (Fig. 2). Subcellular localization studies found that this variant leads to a loss of functional ABCA7 by means of mislocalization from the plasma membrane to the ER [78]. Finally, the largest AD GWAS/GWAX to date, i.e., including AD-by-proxy cases based on parental history of AD, recently identified *ABCA1* as a candidate AD gene [14].

### ABCA2

The *ABCA2* gene is located close to *ABCA1* on chromosome 9q and encodes a 2436 amino acid polypeptide [85, 86]. *ABCA2* mRNA is predominantly expressed in human brain compared to other organs, where it is localized mainly in oligodendrocytes [55, 87]. *ABCA2* mRNA expression in macrophages is upregulated in response to cholesterol influx, classifying *ABCA2* as a sterol-responsive gene [85]. Given its high expression in the brain, a plausible role for ABCA2 in brain lipid homeostasis is hypothesized [88]. Subcellular localization studies in HEK293 cells overexpressing human ABCA2 show high ABCA2 expression in late endosomes/lysosomes, proposing an intracellular lipid trafficking role, rather than transport across the plasma membrane like ABCA1 and ABCA7 [87]. Human ABCA2 expression in Chinese hamster ovary cells leads to the sequestering of LDL-free cholesterol in the lysosome and blocks its delivery to the endoplasmic reticulum (ER) for esterification, mimicking sterol-deprived conditions, and confirming a role in intracellular cholesterol trafficking [89]. Expression of human ABCA2 in HEK293 cells did not significantly alter cholesterol efflux to ApoA1 or ApoE, which again might reflect the endolysosomal location of ABCA2 [90]. However, later research found a decrease in total and membrane cholesterol levels as well as a reduced cholesterol efflux to ApoE ε3 acceptors in mouse neuroblastoma cells expressing human ABCA2, without perturbing lipid rafts [91]. In addition, ABCA2 regulates cholesterol levels by decreasing LDL receptor mRNA and protein expression [91]. The ABCA2 protein expression dynamics in developing rat brain oligodendrocytes coincide with the myelination process, proposing a role for ABCA2 in myelin formation [92, 93]. In fact, *Abca2* knockout mice show abnormal myelin sheet ultrastructure and present with prominent tremor, reduced body weight and hyperactivity, of which the latter two were more prominent in female mice [94]. A second study also observed a tremor in *Abca2* knockout mice, and identified alterations in brain sphingolipid levels, but could not confirm abnormal myelin structure [95].

Differential gene expression analysis of HEK293 cells overexpressing human ABCA2 identified several genes involved in the pathogenesis of AD [96]. Increased expression of human ABCA2 is associated with an increase in APP synthesis and amyloidogenic processing via β-secretase [96, 97]. In parallel, in vitro and in vivo depletion of *ABCA2* is associated with a decrease in Aβ production, due to a decreased γ-secretase cleavage of APP [98]. Confocal microscopy identified colocalization of ABCA2 with both Aβ and APP in intracellular vesicles in human neuroblastoma cells [96]. Of note, the endosomal/lysosomal pathway, showing high levels of ABCA2 expression, is the major site for Aβ generation [99]. *ABCA2* mRNA expression is also significantly increased in brain tissue of the prefrontal cortex and blood of AD patients compared to control individuals [100]. These data suggest a link between elevated ABCA2 expression and AD, and studies clarifying the exact function of the transporter might shed more light on the use of ABCA2 downregulation as a potential therapeutic option for AD.

Methylation of *ABCA2* CpG site cg03349123 is negatively associated with AD risk [100]. Macé and colleagues reported a significant association of a synonymous SNP (rs908832) within exon 14, with EOAD in a French case–control cohort [101]. This association was later confirmed in a Swiss cohort, while in a Greek cohort, the minor allele was significantly more frequent in the control group [102]. Furthermore, rs908832 was monomorphic in a Japanese cohort, suggesting an ethnicity-dependent association with AD [102]. In a Caucasian-American cohort, no significant association was observed either with EOAD or LOAD [103]. Meta-analysis comprising these studies found a strong association of rs908832 with AD (OR = 1.55, 95% CI = 1.12–2.16, *P* = 0.009) [104]. However, rs908832 is not associated with serum cholesterol profiles, and further research is needed to clarify the underlying molecular mechanism this SNP [102].

### ABCA5

*ABCA5* is part of the ABCA gene cluster on chromosome 17q24 [105]. Macrophages from *Abca5* knockout mice show a decreased cholesterol efflux to HDL, identifying *Abca5* as a sterol-responsive gene [106]. A compensatory role for ABCA5 was identified in macrophages after ABCA1 downregulation under hyper-cholesterol conditions [107]. High *ABCA5* mRNA expression is found in the human and mouse brain, where it is predominantly present in neurons [106, 108]. Subcellularly, murine Abca5 localizes in lysosomes and late endosomes as well as at the plasma membrane [107, 109]. *Abca5* knockout mice present with trembling and lysosomal disease-like symptoms such as heart abnormalities, although no abnormalities were detected in brain [109]. ABCA5 is highly expressed in human skin and hair follicles and bi-allelic loss-of-function mutations in *ABCA5* are linked to excessive hair overgrowth (hypertrichosis) [22]. These mutations lead to a disturbed lysosomal function, resulting in the accumulation of autophagosomes and of free cholesterol in endolysosomes in patient hair follicles [22]. A first link with neurodegenerative diseases was established when four SNPs in *ABCA5* were associated with a reduced risk for Parkinson’s disease (PD) in GWAS [110]. In fact, *ABCA5* mRNA expression is significantly increased in PD patient brains [108]. Similar to *ABCA1*, *ABCA5* mRNA expression is significantly elevated in the hippocampus of AD patient brains [106, 111]. Increased expression of human *ABCA5 *in vitro significantly reduces Aβ load, mediated by changes in the processing of APP, suggesting a potential protective role for ABCA5 in AD [106].

### ABCA7

#### Lipid metabolism

Like ABCA1, ABCA7 transfected in vitro mediates plasma HDL formation by releasing cholesterol and phospholipids to ApoA1, the major apolipoprotein in the blood. However, the relative release of cholesterol to phospholipids is much lower than for ABCA1 [112–115]. Recent research showed an increase in ATPase activity of purified ABCA7 in the presence of ApoA1 and ApoE, suggesting a direct interaction between ABCA7 and apolipoproteins [83]. Interestingly, an isoform-dependent stimulation was seen for ApoE, with both ApoE ε4 and ApoE ε2 resulting in a weaker ATPase stimulation compared to ApoE ε3, despite their opposite effects on AD risk, suggesting distinct binding efficiencies between ABCA7 and ApoE isoforms [83]. Additionally, *Abca7* knockout mice showed lower serum and HDL cholesterol levels than wild type mice but only in females, despite the fact that ApoA1-stimulated lipid efflux from macrophages did not differ between wild type and *Abca7* knockout mice [116]. Likewise, suppression of endogenous *Abca7* mRNA by siRNA in mouse fibroblasts did not influence ApoA1-mediated cellular lipid release [117]. In contrast to ABCA1, ABCA7 gene and protein expression is downregulated in BALB/3T3 cells by increased cellular cholesterol and upregulated by cholesterol depletion via the sterol regulatory element-binding protein 2 (SREBP2) pathway [118]. Despite the high homology between ABCA1 and ABCA7, the latter does not compensate for the loss of ABCA1 in Tangier disease patients, further questioning the exact role of ABCA7 in cellular cholesterol release [119]. Moreover, thymocytes and antigen-presenting cells from *Abca7* knockout mice show a reduced trafficking of CD1d, a glycoprotein that presents lipid antigens to natural-killer T (NKT) cells, to the plasma membrane as well as a reduction in lipid rafts, leading to a decreased CD1d content within lipid rafts and subsequently a reduction in NKT cell activation [120]. The role of ABCA1 in NKT cell development has not been studied yet.

#### Phagocytosis

Besides its role in lipid regulation, ABCA7 is functionally linked with phagocytosis. This hypothesis is based on the sequence similarity of ABCA1 and ABCA7 with *ced-7*, a *C. elegans* gene involved in the engulfment of cell corpses during programmed cell death [121, 122]. Indeed, Iwamoto and colleagues found that suppression of *Abca7* with siRNA in mouse fibroblasts results in impaired phagocytic activity, while lipid release is not influenced [118]. Both in vitro and in vivo studies show abolished phagocytosis in *Abca7* deficient mouse macrophages, while phagocytosis was not altered in *ABCA1* deficient cells [117, 122]. Decreasing cellular lipid content using statins was found to enhance phagocytosis via upregulation of ABCA7 in vitro and in mice, indicating a direct link between cholesterol homeostasis and phagocytosis [123]. Mechanistically, ABCA7 and low-density lipoprotein receptor–related protein 1 (LRP1) colocalize at the cell surface of macrophages after stimulation by apoptotic cells. Interaction of LRP1 with apoptotic cells induces extracellular signal–regulated kinase (ERK) signaling, which is required for phagocytosis. *Abca7*-deficiency abolishes ERK signaling by a decreased trafficking of ABCA7 and LRP1 to the cell surface [122]. Of note, LRP1 also interacts with APP to regulate its processing and subsequent Aβ production [124]. Microglia from heterozygous *Abca7* knockout AD mice show an abnormal accumulation of Aβ, likely due to a disturbed endosomal-lysosomal trafficking [125]. These data indicate that, unlike its closest homolog ABCA1, the primary function of ABCA7 is more likely in phagocytosis than in HDL metabolism. In fact, highest *ABCA7* gene expression in primary human brain cells is found in microglia, consistent with a role in phagocytosis [55].

### Functional evidence for a role in AD

Investigation of the cognitive phenotype of *Abca7* knockout mice showed sex-specific differences, revealing impaired novel object recognition in males and impaired spatial reference memory in females [126]. Both in vitro and mice studies show that ABCA7 deficiency results in an increased Aβ load, which can be explained by changes in APP processing [127–129] and/or a reduction in phagocytosis of Aβ by microglia and macrophages [130, 131]. Conversely, AD mice overexpressing ABCA7 show a reduced Aβ expression compared to control AD mice expressing only endogenous ABCA7, as well as an improvement in cognitive function [129]. Expression of murine ABCA7 was also identified in an in vitro BBB model, consisting of primary mouse brain capillary endothelial cell cultures, and was found to regulate the efflux of cholesterol as well as Aβ across the BBB [132]. In addition, primary cultured mice hippocampal neurons show a reduced cell viability and activation of ER stress in the presence of Aβ_1-42_, while overexpression of ABCA7 eliminates these effects [129]. A recent study by Lyssenko and colleagues found reduced ABCA7 protein levels in individuals who developed AD neuropathology at a younger age compared to those who develop it at a later age [133]. These data provide evidence that ABCA7 plays a protective role against AD.

### Genetics of ABCA7

*ABCA7* was cloned from human macrophages in the year 2000 [84]. A decade later, *ABCA7* was first linked to a human disease after GWAS found a strong association between common SNPs in *ABCA7* and LOAD in Caucasian and African American (AA) cohorts [15, 28, 134–136]. Resequencing studies of *ABCA7* in AD patient and control cohorts identified a significant enrichment in patients of rare (minor allele frequency (MAF) ≤ 1%) heterozygous variants, predicted to lead to a PTC. However, multiple studies in Caucasian cohorts show that the association of PTC mutations with AD is independent of the GWAS hits initially identified [79–81, 137–139]. Rare PTC mutations include nonsense, frameshift and splice site mutations as well as the noncanonical c.5570 + 5G > C mutation, which leads to a stop codon due to aberrant splicing of exon 41, and are found in up to 5% of the Caucasian AD patients [140]. Instead, in African Americans, a PTC mutation (rs142076058; p.R587fs), which explains an AA-specific GWAS hit, was identified in over 15% of the patients [141]. In addition, CpG methylation in the *ABCA7* locus is significantly associated with AD [142].

PTC mutations are expected to lead to a loss-of-function due to nonsense-mediated decay (NMD), a quality control mechanism that removes transcripts containing a premature stop codon. However, *ABCA7* transcript analysis revealed not only escape from NMD, but also transcript rescue, with high variability between the different PTC mutations [80]. Further, a strong association was identified between a GWAS hit and a variable number tandem repeat (VNTR) in intron 18 [143]. Longer VNTR alleles are enriched in AD patients and correlate with a decrease in overall *ABCA7* expression and an increase in alternative splicing leading to in-frame skipping of exon 19, which results in a partial loss of the first NBD [143]. Moreover, rare missense mutations are enriched in patients compared to healthy controls [142]. Recent work shows that predicted deleterious *ABCA7* missense mutations cause subcellular protein mislocalization as demonstrated in HeLa cells. Wild type ABCA7 is predominantly expressed at the plasma membrane [78, 113]. However, the mutated protein is not able to reach the plasma membrane and is retained in the ER instead, resulting in a loss of functional protein [78]. Not surprisingly, these findings are in line with data from ABCA1, 3 and 4. Disease-associated missense mutations in these *ABCA* genes are found to lead to protein mislocalization or to impact the function of the protein by abolishing substrate binding and/or ATPase activity [144–146]. Interestingly, functional and subcellular localization studies on disease-causing *ABCA1* mutations and their corresponding *ABCA4* mutations show similar effects, implying a comparable structure–function relationship [147]. Consequently, pathogenic missense mutations in *ABCA1*, *3*, *4, 5* and *12*, which are implicated in human diseases, might pinpoint amino acid residues that are essential for the correct functioning of ABCA transporters in general. Alignment of the ABCA amino acid sequences shows that several published *ABCA7* missense mutations affect amino acid residues which are conserved between ABCA7 and ABCA1, 4 and 12 and that these residues, when mutated in ABCA1, 4 and 12, are disease-causing (Fig. 2). Additionally, a protective effect is observed for a common *ABCA7* missense variant (rs72973581; p.G215S), suggesting a bidirectional effect of missense mutations [82]. A high-throughput assay to assess the functional impact of *ABCA7* variants is currently not available. Characterization of subcellular localization and functional effects of *ABCA7* missense mutations is needed to discriminate pathogenic variants from neutral or protective variants. Recently, a cryo-electron microscopy structure of ABCA7 was announced (PDB ID: 7KQC) which will advance our understanding of the mechanistic consequences of missense mutations [83].

Bi-allelic mutations i.e., homozygous or compound heterozygous mutations, in *ABCA1*, *3*, *4*, *5* and *12* are implicated in recessive disorders. Monoallelic mutations are linked to increased disease risk or milder phenotypes [54, 148, 149]. Earlier, we explored the role of rare homozygous and compound heterozygous mutations in *ABCA7* and identified *cis* and *trans* compound heterozygous mutations but no rare homozygous mutations [78]. Although presently understudied, deep-intronic and common variants in *ABCA4* play a role in disease as well and can contribute to recessive inheritance [150, 151]. Likewise, investigating the contribution of common and noncoding variants in *ABCA7* is important since they might influence disease risk or act as a modifier in carriers of a PTC or deleterious missense mutation.

### Clinicopathological phenotype of *ABCA7* mutation carriers

*ABCA7* was initially associated with LOAD in GWAS, while PTC mutations are enriched in both LOAD and EOAD patients [79–81]. PTC carriers present with a very wide range in onset age of 46–90 years old [81, 140]. Onset age is independent of the type of PTC mutation, since variable onset age is observed between carriers of the same PTC mutation, suggesting the involvement of modifying factors [140]. However, presence of the *APOE* ε4 allele does not have a significant impact on onset age in these carriers [140]. Investigation of larger cohorts of carriers is of high importance to study the co-occurrence with *APOE* isoforms or other potential genetic and environmental modifiers [140]. Besides the possible presence of genetic modifiers, NMD-efficiency and ABCA7 protein expression may play a role in age-related penetrance of PTC mutations. In fact, NMD efficiency and protein expression differ between individual PTC mutations, whereas transcript rescue differs even between carriers of the same PTC mutation [80]. Carriers of an expanded VNTR allele also present with widely variable onset ages, ranging from 44–90 years old, although no association is observed between VNTR length and onset age [143].

In PTC mutation carriers, clear clinically defining characteristics are absent, and most carriers present with a classical amnestic AD phenotype albeit with a higher vascular involvement (Hendrickx Van de Craen et al., unpublished) [152]. Brain autopsy of 10 PTC mutation carriers (five frameshift, three splice and two nonsense mutations) and six missense mutation carriers shows hallmark AD pathology (i.e., senile plaques and neurofibrillary tangles), as well as CAA in all carriers plus capillary (type 1) CAA in all but one nonsense mutation (p.W1336*) carrier (Hendrickx Van de Craen et al., unpublished) [78, 152]. A genetic association study exploring the association of AD risk loci with AD neuropathological features in 256 participants aged ≥ 85 years identified a significant association between the *ABCA7* locus and Braak stage as well as CAA, but not capillary CAA [153]. Cerebrospinal fluid biomarker analysis revealed a negative correlation between Aβ1–42 and VNTR length [143].

Familial clustering is higher in PTC carriers compared to the overall AD cohort, with a positive familial history found in up to 77% of PTC carriers [80, 140, 152]. Apparent co-segregation of missense mutations (p.R880Q, p.G1820S) [78, 154] and PTC mutations (p.E709fs, p.R578fs, c.3578-2A > C and p.L1043fs) [138, 141, 154, 155] has been described in six pedigrees, all mimicking an autosomal dominant inheritance pattern. However, co-segregation in these pedigrees is not significant, because the pedigrees are too small to reach a significant logarithm of the odds (LOD) score. Zhao and colleagues developed a rare variant non-parametric linkage analysis method to detect rare variants contributing to complex diseases segregating in families and applied this method to whole genome sequencing data of 107 LOAD pedigrees with Caribbean Hispanic and European ancestry [156]. Nominal significant linkage was observed for *ABCA7*, with 13 rare missense variants segregating in 20 Caribbean Hispanic families [156].

The increased disease risk and familial clustering associated with *ABCA7* PTC mutations suggest that these carriers might represent a genetic subtype of AD. In the future, screening of *ABCA7* PTC mutations in clinical practice might improve AD diagnosis and risk prediction, mainly in familial AD patients negative for mutations in *APP*, *PSEN1* and *PSEN2*. However, further research is necessary to elucidate both the pathogenicity and the disease penetrance of individual *ABCA7* PTC mutations.

## Conclusions

Functional studies suggest a central role for ABCA transporters in the maintenance of the cholesterol-homeostasis in the brain. Dysregulation of these transporters might result in cholesterol accumulation, leading to toxic effects and neurodegeneration. *ABCA7* was first associated with AD by GWAS. Resequencing studies have identified an enrichment of PTC mutations, missense mutations and an expanded VNTR in AD patients. Recently, *ABCA1* was also linked to AD in GWAS, although the underlying functional variants are currently unknown. In total, genetic and functional studies link four members of the ABCA subfamily (ABCA1, 2, 5 and 7) to AD. All four members were found to modulate Aβ deposition, one of the neuropathological hallmarks of AD, albeit in different directions. Disease-modelling using patient-derived iPSCs can allow to identify and study the cellular mechanisms disturbed by mutations in *ABCA* genes in human brain cells. A better understanding of the function of ABCA transporters in physiological and pathophysiological conditions will help to better understand their role in the etiopathogenesis of AD and might aid the development of new therapeutic strategies as well as contribute to the genetic diagnosis of AD.

## Data Availability

Not applicable.

## Abbreviations

AA: African American
ABCA: ATP-binding cassette transporter member A
ABC: ATP-binding cassette transporter
AD: Alzheimer’s disease
APOE: Apolipoprotein E
Aβ: Amyloid β
APP: Amyloid precursor protein
BBB: Blood brain barrier
CAA: Cerebral amyloid angiopathy
EOAD: Early-onset Alzheimer’s disease
ERK: Extracellular signal–regulated kinase
GWAS: Genome-wide association study
HDL: High-density lipoprotein
LDL: Low-density lipoprotein
LOAD: Late-onset Alzheimer’s disease
LRP1: Low-density lipoprotein receptor–related protein 1
LXR: Liver X receptor
NBD: Nucleotide binding domain
NKT: Natural-killer T cell
NMD: Nonsense-mediated mRNA decay
PSEN1/2: Presenilin 1/2
PTC: Premature termination codon
SNP: Single nucleotide polymorphism
SREBP2: Sterol regulatory element-binding protein 2
TD: Tangier disease
TMD: Transmembrane domain
VNTR: Variable number tandem repeat
